# Role of Tumor Necrosis Factor in Tuberculosis

**DOI:** 10.3390/biom15050709

**Published:** 2025-05-12

**Authors:** Fedor D. Kireev, Julia A. Lopatnikova, Alina A. Alshevskaya, Sergey V. Sennikov

**Affiliations:** 1 Laboratory of Molecular Immunology, Federal State Budgetary Scientific Institution “Research Institute of Fundamental and Clinical Immunology” (RIFCI), 630099 Novosibirsk, Russia; f.kireev@mail.ru (F.D.K.); lopatnikova18@yandex.ru (J.A.L.); 2 Laboratory of Immune Engineering, Federal State Autonomous Educational Institution of Higher Education “I.M. Sechenov First Moscow State Medical University” under the Ministry of Health of the Russian Federation (Sechenov University), 119048 Moscow, Russia; alkkina@yandex.ru

**Keywords:** TNF, TNFR1, TNFR2, Tuberculosis, Anti-TNF therapy

## Abstract

Tumor necrosis factor (TNF) is a key immunoregulatory cytokine with a dual role in the host response to *Mycobacterium tuberculosis*. While essential for granuloma formation, macrophage activation, and containment of latent infection, TNF can also contribute to tissue damage and immune pathology. This review systematically analyzes over 300 peer-reviewed studies published between 1980 and 2024, highlighting the molecular and cellular mechanisms of TNF action in tuberculosis (TB). Particular attention is given to TNF receptor signaling pathways, the balance between protective and pathological immune responses, and the modulation of TNF activity during anti-TNF therapy in patients with autoimmune diseases. We discuss how different TNF inhibitors vary in their capacity to interfere with host defense mechanisms, with monoclonal antibodies carrying a higher reactivation risk than receptor-based agents. To enhance conceptual clarity, we provide newly developed schematic representations that integrate current knowledge on TNF-driven immune dynamics, including its interaction with other cytokines, effects on granuloma stability, and role in intracellular bacterial control. Understanding the pleiotropic functions of TNF in tuberculosis pathogenesis is crucial for developing safe immunomodulatory strategies and optimizing the clinical management of patients at risk of latent TB reactivation.

## 1. Introduction

Tuberculosis (TB) is a bacterial infectious disease with predominant lung damage and high mortality. *Mycobacterium tuberculosis* (*M. tuberculosis*) is the causative agent of TB. It is an intracellular bacterium that infects and persists in macrophages and other myeloid cells. TB is one of the ten leading causes of death and the main leading cause of mortality from a single infectious agent. There are 1.7 billion people latently infected by *M. tuberculosis* worldwide; about 10 million people fall ill and 1.2–1.4 million die from TB annually in the world [1]. Active TB develops in 5–10% of infected individuals, and latent TB infection develops in most infected individuals, which can continue throughout life [2,3,4,5]. TB is the main cause of death in patients with HIV (0.3 million people per year) [1].

**Figure 1:**
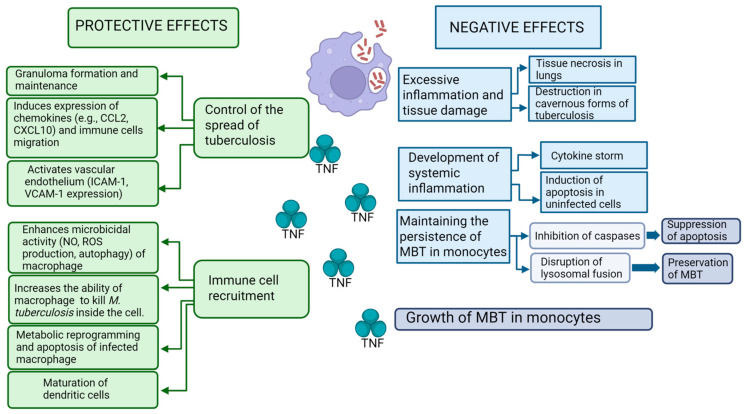
Protective and negative effects of TNF during *M. tuberculosis* infection. Created in https://BioRender.com.

The pro-inflammatory cytokine tumor necrosis factor (TNF) plays a key role in the immune response against *M. tuberculosis*. During the immune response, TNF performs a number of important functions: apoptosis of infected macrophages, activation of macrophages with the formation of reactive oxygen species and nitrogen species, maturation of dendritic cells with subsequent activation of T cells, and secretion of interferon-gamma (IFN-γ), stimulation of the chemotaxis of T lymphocytes and monocytes, and granuloma formation. At the same time, TNF can also have a negative effect on the course of TB infection, mediating granuloma disorganization and pathogen growth, tissue damage, *M. tuberculosis* reproduction in macrophages, and the manifestation of clinical symptoms of the disease [6,7,8] (Figure 1). Understanding protective immunity and immunopathogenesis mechanisms in TB is one of the main tasks of modern phthisiology [6,7,8,9].

## 2. Tumor Necrosis Factor and Its Receptors

TNF is initially synthesized as a transmembrane protein [10,11]. Its membrane form can be cleaved by TNF-α converting enzyme (TACE), producing a biologically active soluble form (17 kDa) [12,13]. TNF signals through two receptors: TNFR1 (55 kDa, with a death domain), broadly expressed, and TNFR2 (75 kDa, without a death domain), which is expressed by immune and stromal cells, including T and B lymphocytes, monocytes, and endothelial cells [14,15,16,17,18,19,20,21,22,23]. The soluble TNF form primarily activates TNFR1, while the membrane form activates both receptors [24,25,26,27,28,29,30]. TNFR1 mediates apoptosis and NF-κB activation, while TNFR2 supports TNFR1 signaling and modulates lymphoid cell proliferation [27,31,32,33,34,35,36,37]. The physiological role of TNFR2 also includes regulatory and tissue-protective functions [32,33,34,35,36,37].

The main TNF-producing cells are monocytes, macrophages, and T lymphocytes [38]; in addition, it is secreted by B lymphocytes [39], mast cells [40], polymorphonuclear leukocytes [41], erythroblasts [42], keratinocytes [43], astrocytes and microglial cells [44], smooth muscle cells [45], Paneth intestinal cells [46], tumor cells and tumor microenvironment cells [38,47], and mesangial cells [48].

In vitro TNF stimulates the proliferation of fibroblasts [49], astrocytes [50], macrophages [51], T-lymphocytes, and B-lymphocytes [52,53,54,55], but has a cytotoxic effect on various tumor cell lines [56] and normal cells, such as endothelial [57,58], oligodendrocytes [59], thyrocytes [60], and fibroblasts [61]. In vivo, the administration of TNF produces manifestations similar to those of endotoxemia [62,63,64,65]. TNF can both directly act on cells and cause mediated effects by inducing the production of other mediators [29,38].

Unregulated TNF production can have a damaging effect—it is associated with various autoimmune and inflammatory diseases, such as rheumatoid arthritis [66], septic shock [67], inflammatory bowel disease [68,69], and multiple sclerosis [70,71]. TNF blockers have been shown to be effective in controlling inflammation associated with rheumatoid arthritis [72], ankylosing spondylitis [73], Crohn’s disease [74], and psoriasis [75]. However, systemic inhibition of TNF production can lead to undesirable side effects due to the weakening of the body’s defenses against pathogens; for example, there are cases of TB reactivation in patients with rheumatoid arthritis treated with TNF blockers [76]. Since in vivo TNF is produced by many cell types [77,78], cell-specific TNF production can be one of the mechanisms by which the balance between protective and damaging mediator functions is regulated [79].

Soluble TNF type 1 and type 2 receptors are identical to the extracellular cytokine-binding domains of TNF membrane receptors [80,81,82,83,84]. They can compete with membrane-bound receptors for binding to TNF and thus block its biological activity [80,81,82,83]. Soluble TNF receptors are formed by shedding with TACE metalloproteinase (ADAM-17), which is also responsible for the formation of the soluble form of TNF, the extracellular domains of the corresponding membrane receptors [13,85]. It has been found that soluble TNFR2 can be formed [86,87,88,89,90] through an alternative splicing mechanism involved in the formation of polymorphic variants of cytokines and their membrane and soluble receptors, which may have different biological activities [91], both in healthy individuals and in various pathologies [86,87,88,89,90]. Soluble TNFR1 can also be formed by alternative splicing, but these forms are associated with autoimmune diseases [92,93].

Soluble TNF receptors of both types are present in the serum of healthy individuals at concentrations of approximately 0.7 ± 0.2 ng/mL for TNFR1 and 2.2 ± 0.4 ng/mL for TNFR2, independent of age and sex [94,95]. TNF production can be stimulated by several cytokines and growth factors, including GM-CSF, IL-3 [96], IFN-γ [97], IL-2 [98], IL-6 [99], IL-17 [100], IL-1, and TNF itself [101,102]. T lymphocyte activation through antigen recognition is also a key in vivo mechanism of TNF upregulation [103,104], which is commonly observed in autoimmune and infectious diseases [105,106,107,108,109].

**Figure 2:**
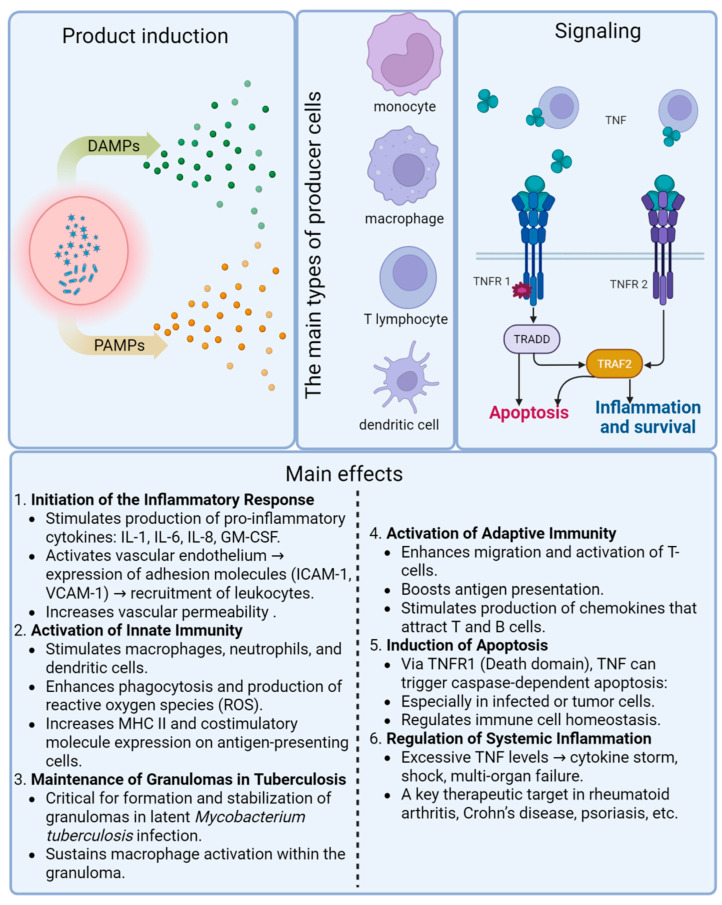
Mechanisms of TNF production, signaling pathways, and major immunological effects during *M. tuberculosis* infection.

Additionally, TNF—like other members of its superfamily—can mediate reverse signaling, in which the signal is transmitted from the receptor or antibody back to the membrane-bound ligand [110,111]. The membrane form of TNF can be activated by both soluble and membrane-bound TNF receptors [111]. The main mechanisms of TNF production, receptor-mediated signaling, and functional roles during TB infection are illustrated in Figure 2.

### Tumor Necrosis Factor and Its Receptors in Tuberculosis

TNF receptors have similarities and differences in mediated functions in TB infection. The type 1 receptor plays a major role in resistance to *M. tuberculosis* through the induction of pro-inflammatory and proapoptotic responses, while the type 2 receptor is more effective (in membrane and soluble forms) as a TNF neutralizer [6,9].

TNF and its type 1 receptor are required to control *M. tuberculosis* infection [9]. Thus, it was shown in a mouse model that the administration of neutralizing monoclonal antibodies against TNF leads to the rapid development of *M. tuberculosis* infection and death of mice. At the same time, the number of live mycobacteria of TB in the spleen, liver, and lungs in mice injected with neutralizing anti-TNF antibodies was 10–100 times higher compared to mice injected with IgG [9]. The development of lethal *M. tuberculosis* infection was shown in the experiments with TNFR1 knockout mice. In the organs of knockout mice, the number of pathogens was 10–50 times higher compared to animals with TNF receptor type 1 [9].

## 3. Levels of TNF and Its Membrane and Soluble Receptors in Tuberculosis

### 3.1. Levels of TNF and Its Soluble Receptors in Tuberculosis

The effective execution of TNF-mediated functions in host defense requires its production at precise anatomical sites, at appropriate times, and in physiologically relevant concentrations. Numerous studies have investigated circulating TNF levels in individuals with TB, yielding heterogeneous results. While the majority of reports indicate elevated TNF levels in patients with active disease compared to healthy individuals [112,113,114], several studies have not found statistically significant differences [115,116,117]. This inconsistency may reflect the compartmentalization of cytokine responses in TB, with locally elevated levels at the site of infection and relatively suppressed systemic concentrations [118]. Additionally, TNF levels appear to correlate with disease severity: patients with advanced or disseminated forms of TB typically exhibit higher cytokine concentrations than those with localized pulmonary involvement [117,119]. Variability in TNF expression is also associated with distinct pathological manifestations. For example, enhanced *TNF* gene expression has been demonstrated in necrotizing tuberculomas, in the fibrotic walls of cavities characteristic of fibro-cavernous TB, and adjacent lung parenchyma not overtly affected by infection [120]. These findings underscore the dual nature of TNF in TB pathogenesis: while it plays a critical role in orchestrating protective immunity, it is also implicated in driving immunopathology. Notably, TNF levels tend to decline following anti-tuberculosis therapy [112], and geographic and population-based factors may contribute to inter-individual differences in cytokine profiles [114]. 

*M. tuberculosis* strains differ in their ability to modulate host cytokine responses. Variants with higher virulence tend to induce stronger TNF production, while less virulent strains are associated with reduced TNF and IL-12 secretion, and in some cases, increased IL-10 levels [121,122,123,124,125,126,127,128]. These differences may contribute to immune evasion and persistent infection.

In patients with pulmonary TB with increased content of serum TNF, the levels of its soluble type 1 and 2 receptors were also increased [129]. Whereas other studies show that the levels of soluble types 1 and 2 receptors in the blood serum in TB patients were lower in comparison with healthy donors. This is probably due to the absence of an increased systemic level of the cytokine itself in the blood serum in patients with TB [117]. At the same time, like TNF levels, the levels of soluble cytokine type 1 and 2 receptors in patients with an advanced infectious process are increased compared to patients with a limited infectious process [117]. This increase appears to be a regulatory mechanism for neutralizing high levels of TNF to prevent granuloma disorganization and tissue damage [130,131]. Thus, the level of soluble TNF receptors in pulmonary TB depends on the volume of damage to the lung tissue, the type of *M. tuberculosis* strain that caused the infection, and the pathoanatomical picture of the disease.

### 3.2. Expression of Type 1 and Type 2 TNF Membrane Receptors in Tuberculosis

In tuberculous granulomas, the highest expression of TNF receptors type 1 and type 2 is observed on dendritic cells, macrophages, and B cells, with TNFR1 expression exceeding that of TNFR2 [19]. In the peripheral blood of patients with active pulmonary TB, the percentage of TNFR1^+^ T cells, B cells, and TNFR2^+^ B cells is increased compared to healthy individuals. Moreover, the number of TNFR1 molecules per cell is elevated in T cells and monocytes, while increased TNFR2 expression is observed only in monocytes [18].

These findings indicate that TB is associated with both quantitative and qualitative changes in TNF receptor expression, particularly with a predominance of TNFR1, consistent with its central role in mediating protective immunity against *M. tuberculosis*.

Evidence for the function of TNF and its receptors in TB has been obtained from studies in both patients and experimental models. Notably, the main immunological features, including the protective role of TNF, are broadly conserved between humans and laboratory animals [132,133,134,135,136].

## 4. Stimulation of TNF Production by Mycobacterial Components

Multiple immune cell types contribute to TNF production in TB, including monocytes, macrophages, dendritic cells, neutrophils, and various T cell subsets—such as CD4^+^, CD8^+^, γδ T cells, NKT cells, and CD1-/MR1-restricted T cells [38,137,138,139,140,141,142,143,144,145]. The frequency of *M. tuberculosis*-specific TNF-producing T cells may also assist in distinguishing latent from active TB [142].

Lipoarabinomannan (LAM), a glycolipid of *M. tuberculosis*, has a pronounced stimulating effect on the production of TNF [146]. In addition, 30 kDa (Ag85B) and 38 kDa antigens, as well as the EsxL antigen of the pathogen, also enhance TNF production [147,148,149]. Toll-like receptors (TLRs), which are mostly extracellular receptors of innate immunity, participate in the regulation of TNF production by macrophages, which play an important role in the immune response in TB infection: in TLR2- and TLR4-deficient mice, the infection proceeds more severely, and death occurs faster due to the absence of TNF secretion by macrophages [150]. *M. tuberculosis* DNA binds to TLR9 and causes an increase in TNF production by M1 macrophages, but not by M2 macrophages [151]. Adaptor proteins of TLR MyD88 and TRIF signaling pathways are also important for the production of pro-inflammatory cytokines and the protective immune response in *M. tuberculosis* infection: in mice deficient in MyD88 and TRIF upon contact with the pathogen, TNF production is absent or reduced [152,153,154,155]. Lectin C-type receptors are also involved in stimulating TNF production [156,157], but after binding with mannose-capped LAM, not only an increase in the production of pro-inflammatory mediators TNF and IL-6 occurs, but also an increase in the production of immunosuppressive cytokines IL-10 and TGF-β, which can inhibit TNF production [156,157]. NOD receptors, which are intracellular receptors of innate immunity, are also involved in stimulating TNF production [158,159]. In addition, TNF can promote the reproduction of *M. tuberculosis* in newly infected macrophages [160], which leads to increased Ag85B secretion and further stimulation of TNF production [147]. *M. tuberculosis* lipomannans, through interaction with TLR2, cause expression of microRNA 125b in macrophages, which leads to degradation of TNF mRNA and, accordingly, to a decrease in mediator production [161]. TNF itself and a number of cytokines can stimulate the production of this mediator, while other cytokines have an inhibitory effect on the production of TNF. Thus, IFN-γ produced by alveolar macrophages [162] and T lymphocytes [163] can enhance macrophage production of TNF [164]. In contrast to TLRs and NOD receptors, certain C-type lectin receptors can inhibit TNF production. For example, DC-SIGN, expressed on dendritic cells and alveolar macrophages during TB infection, is utilized by *M. tuberculosis* for cell entry via mannose-capped lipoarabinomannan [165]. Signaling through DC-SIGN antagonizes TLR-mediated pathways by promoting NF-κB acetylation and enhancing IL-10 secretion, ultimately suppressing TNF production [166].

## 5. Protective Effects of TNF During Tuberculosis Infection

### 5.1. Role of TNF in Apoptosis of M. tuberculosis-Infected Macrophages

Five types of cell death contribute to the outcome of *M. tuberculosis* infection, including apoptosis, necrosis [167], autophagy [168], pyroptosis [169], and ferroptosis [170,171]. Apoptosis and autophagy are generally associated with protective immunity, as they contain the pathogen and facilitate antigen presentation. In contrast, necrosis and other non-programmed forms of death often promote bacterial dissemination. TNF regulates macrophage death in TB through both apoptosis and necrosis, with apoptosis playing a particularly important role in pathogen elimination by limiting mycobacterial viability and promoting antigen presentation [172]. During apoptosis, infected macrophages generate apoptotic blebs containing mycobacterial antigens, which can be engulfed by uninfected antigen-presenting cells for cross-presentation via MHC I and CD1 pathways [168,169,170]. This alternative way of CD8 T cell activation has great immunopathogenetic importance since pathogen antigens contained in the phagosome do not penetrate the cytoplasm and cannot be processed in the classical way with further presentation in complex with MHC I. It has been shown that macrophages immediately after infection with *M. tuberculosis,* lose the ability to present mycobacterial antigens and activate CD8 T cells [173]. Furthermore, CD1 expression on dendritic cells decreases when infected with mycobacteria [174]. While uninfected dendritic cells express high levels of MHC, CD1, and co-stimulatory molecules for T cell priming [175]. Activated CD8 T cells, in turn, secrete IFN-γ, which activates uninfected macrophages and, therefore, is of key importance in controlling mycobacterial proliferation [176]. IL-10, which stimulates the expression of the anti-apoptotic Bcl-2 protein [177], is an antagonist of TNF for apoptosis induction [177,178]. In addition, IL-10 stimulates TNFR2 shedding, which leads to the binding of the mediator and inhibition of its biological activity, including induction of apoptosis [179]. Since IL-10 is an antagonist of TNF for the induction of macrophage apoptosis, the ratio of the effects of these mediators plays an important role in the outcome of the infectious process [180]. Despite the fact that virulent and avirulent strains of this pathogen cause the production of comparable levels of TNF, avirulent strains are stronger inducers of apoptosis compared to virulent ones [181]. This is probably due to the fact that virulent strains enhance type 2 TNF receptor shedding, which leads to TNF binding and inhibition of its effects [179]. It was shown that deficiency of caspase-8 and pro-apoptotic Bcl-2 proteins BAX and BAK in myeloid cells, which are required for TNF-induced apoptosis, led to increased inflammation and increased bacterial load [182]. Accordingly, pharmacological suppression of apoptosis inhibitor proteins (IAP), which leads to the blocking of cell survival signals during signal transduction from TNF via its type 1 receptor, contributes to apoptosis strengthening of infected macrophages and a decrease in the bacterial load [182].

### 5.2. Role of TNF in the Immunometabolism of Macrophages Infected by M. tuberculosis

In their resting state, alveolar macrophages rely primarily on oxidative phosphorylation and fatty acid oxidation for energy production. However, upon infection with *M. tuberculosis*, a metabolic shift toward aerobic glycolysis and increased lactate production occurs, which supports intracellular control of the pathogen [183]. Similar metabolic reprogramming is observed in macrophages stimulated with lipopolysaccharide (LPS), where enhanced glycolysis is associated with increased production of TNF and IL-1β, although the regulatory pathways involved may differ [184]. In *Mycobacterium bovis bacillus Calmette-Guerin* (BCG) infection, macrophages also upregulate glycolysis in parallel with TNF and IL-1β secretion [185]. The end product of glycolysis, lactate, contributes to *M. tuberculosis* clearance, in part by inducing autophagy [186], but it also exerts an anti-inflammatory effect by downregulating TNF and IL-1β production [186]. Thus, *M. tuberculosis* infection induces TNF expression alongside glycolytic reprogramming, and lactate plays a dual role by enhancing antimicrobial defense while dampening excessive inflammation.

### 5.3. Role of TNF in the Maturation of Dendritic Cells

Dendritic cells are essential for initiating the T cell-mediated immune response during *M. tuberculosis* infection, as they are the most efficient antigen-presenting cells for presenting bacterial antigens to T cells. They are able to capture *M. tuberculosis* in the focus of infection and migrate to secondary lymphoid organs, in particular, to axillary lymph nodes [187]. In the lungs, dendritic cells are located in the alveolar spaces [188]. Dendritic cells activate CD4^+^ T cells in lymph nodes, initiating their proliferation and cytokine production (primarily IL-2 and IFN-γ), which enhances the antimicrobial activity of monocytes and promotes cytotoxic responses essential for the control of *M. tuberculosis* infection [189]. TNF together with GM-CSF mediates the differentiation of myeloid progenitor cells into dendritic cells and macrophages [190]. In vitro studies showed that two steps were required for the differentiation of functional dendritic cells [191]. In the first step, the differentiation of monocytes into immature dendritic cells occurs when exposed to TNF and GM-CSF. In the second step, stimulation by bacterial components is needed for their differentiation into highly efficient dendritic cells, which mediate the proliferation of resting CD4+ T cells and their production of cytokines Th1 (IL-2 and IFN-γ) and Th17 (IL-17) [191]. Therefore, TNF is involved in the regulation of the Th1 and Th17 immune response induced by mature dendritic cells, and stimulation of monocytes by TNF takes part in the regulation of the adaptive immune response in TB. However, the prolonged effect of TNF on dendritic cells can also lead to the growth of pathogens, because the ability of bronchoalveolar lavage dendritic cells to capture and kill *M. tuberculosis* compared to immature dendritic cells was shown [192]. In addition, unlike non-virulent BCG [193], virulent *M. tuberculosis* inhibits dendritic cell maturation, and simultaneous exposure to TNF, IL-1β, and prostaglandin E2 is necessary to abrogate this inhibitory effect [194].

### 5.4. TNF Stimulates the Production of Chemokines and Adhesion Molecules

Chemokines and their receptors are responsible for cell migration and localization [195,196,197]. These processes are key for granuloma formation in TB infection [198,199,200,201], and TNF plays an important role in their regulation [198]. TNF stimulates the production of chemokines such as CCL2 (MCP-1) [202,203,204,205], CCL3 (MIP-1α) [206,207], CCL4 (MIP-1β) [207,208,209], CCL5 (RANTES) [210,211,212], CCL7 (MCP-3) [203,204], CCL11 (eotaxin) [213,214], CXCL2 (MIP-2) [215], CXCL-8 (IL-8) [216,217,218], CXCL9 (MIG) [219,220,221], CXCL10 [205,221,222,223], CXCL11 (I-TAC) [219], ICAM-1 adhesion molecules [202], and VCAM-1 [202,224]. An increase in the concentration of chemokines creates chemotactic gradients, which causes the migration of cells from the bloodstream and other parts of the lung to the focus of infection [198,199,200,201]. In addition, TNF stimulates the production of adhesion molecules such as VCAM-1, ICAM-1, and E-selectin, which promotes the migration of activated immune cells into the infectious focus [202,225,226].

### 5.5. Role of TNF in Granuloma Formation and Maintenance

**Figure 3:**
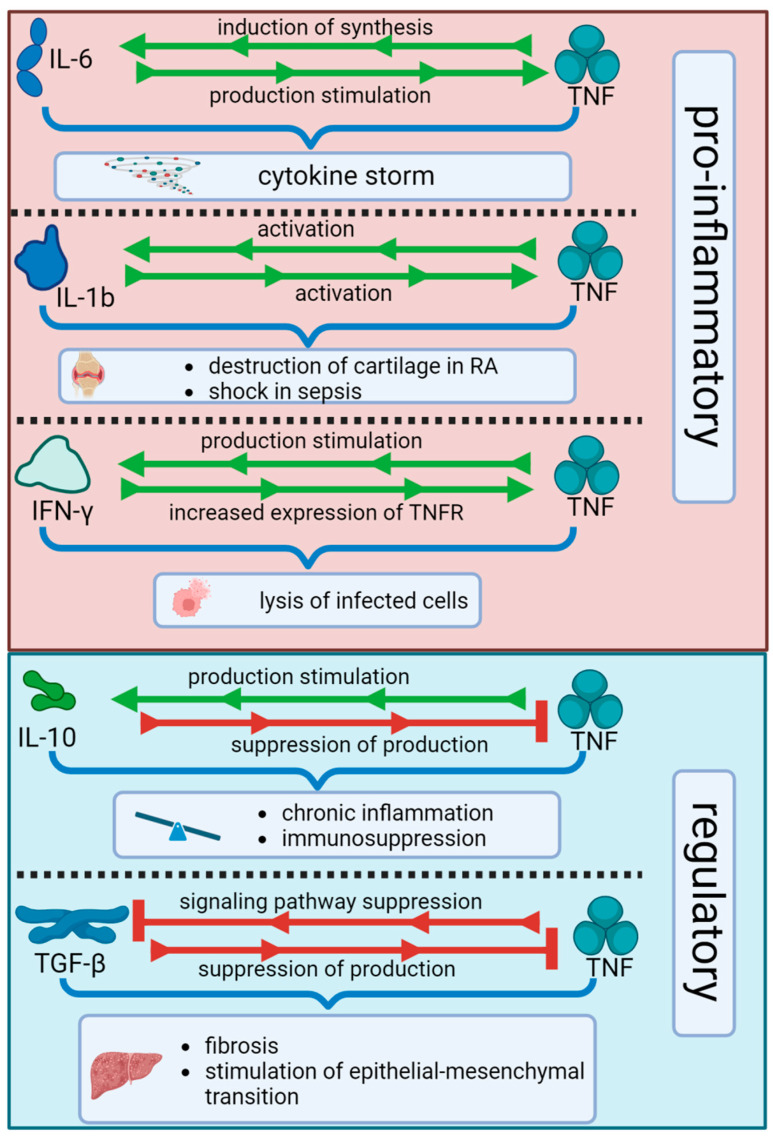
Cytokine interaction network: pro-inflammatory and regulatory interplay with TNF during tuberculosis.

Macrophages are able to limit the growth of mycobacteria as early as 24 h after infection [227]. However, some pathogenic bacteria can survive in macrophages by phagocytizing them, which leads to the formation of a granuloma, the main function of which is to prevent the spread of the pathogen [228]. TNF is a key factor in the formation and maintenance of granuloma structure. Increased expression of TNF, IFN-γ and IFN-γ RNA, as well as TNF-inducible chemokines CXCL9, CXCL10, and CXCL11, which are CXCR3 ligands, was shown in granuloma [229,230]. Migration of CXCR3+ cells, producing IFN-γ, to the granulomatous focus, occurs. Thus, a high level of cytokines IFN-γ, TNF, and the chemokines they induce is maintained in the granuloma, and the cells are in an activated state, which is necessary for granuloma maintenance [230]. TNF is essential for the early induction of chemokines (e.g., CCL2, CCL3, CCL4, CXCL9–11) and for proper spatial organization of macrophages and lymphocytes in granulomas, thereby creating a protective pulmonary microenvironment [231,232]. In TNF-deficient mice, delayed chemokine production, impaired T cell regulation, and disorganized granuloma architecture are observed, while wild-type animals form structurally intact granulomas with timely cytokine responses. The absence of a TNF signal leads to a rapid disruption of the granuloma structure, an increase in its volume, and necrosis of macrophages both inside and outside the granuloma [9,228]. Necrosis of macrophages in the absence of TNF may be due to increased pathogen growth [228]. On the other hand, in TNF deficiency, macrophage death occurs through other mechanisms; for example, through a caspase-independent cell death pathway [233,234], which is triggered by *M. tuberculosis* only at a high bacterial load in macrophages, consistent with both increased pathogen growth and cell necrosis [228]. Increased macrophage death leads to further bacterial growth and tissue destruction. Therefore, one of the key functions of TNF in TB is the survival of macrophages [228]. Thus, disturbances in the formation of granuloma and its structure may be due to its insufficient formation [102,231,235] and its structural instability [9,228]. Figure 1 and Table 1 provide an overview of the dual role of TNF in tuberculosis, including both the protective and pathological consequences of its signaling. The complex interplay between TNF and other cytokines such as IFN-γ, IL-1β, IL-6, IL-10, and TGF-β is presented in Figure 3.

## 6. Anti-TNF Therapy in Autoimmune Diseases and Tuberculosis

### 6.1. Negative Effects of Anti-TNF Therapy on Antituberculous Immunity

**Figure 4:**
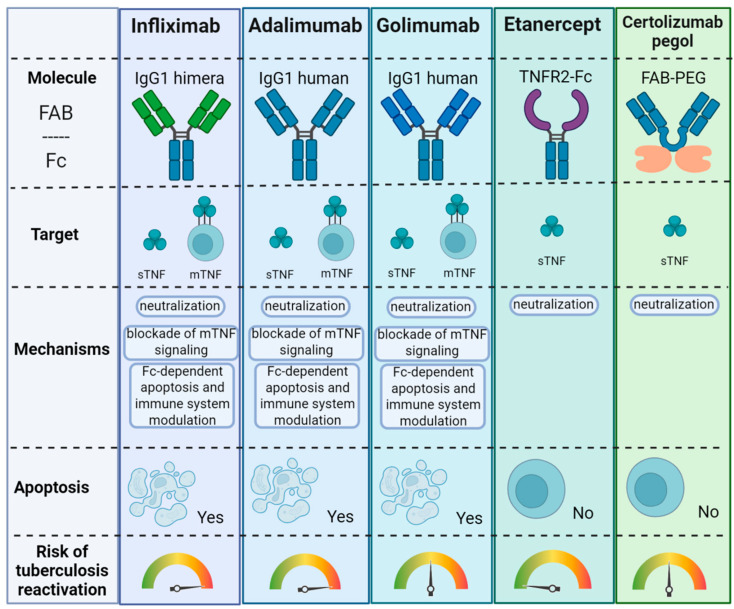
Negative effects of anti-TNF therapy on antituberculous immunity. Created in https://BioRender.com.

TNF is an important pro-inflammatory cytokine, the level of which is increased in many autoimmune diseases; therefore, TNF inhibitors are widely used in the therapy of these nosologies. Thus, TNF blockers are used in rheumatoid arthritis, ankylosing spondylitis, ulcerative colitis, Crohn’s disease, and psoriatic arthritis [236]. However, since TNF plays a key role in the anti-TB immune response [6,7,8], reactivation of latent or even development of primary TB can occur during anti-TNF therapy in patients [6,7,8,237,238,239]. In mice, it has also been shown that animals become more susceptible to *M. tuberculosis* infection when TNF is neutralized [235,240,241]. TNF blockers can reduce the important effects of TNF, which is necessary to protect against TB infection: stimulation of chemokine secretion and expression of adhesion molecules, macrophage apoptosis induction, nitric oxide production, and maintenance of the tuberculous granuloma structure [6,239]. TNF neutralization also leads to the inhibition of IFN-γ effects [242,243,244] and the increased apoptosis of monocytes [245], CD4+ T helpers [246,247] and *M. tuberculosis*-specific CD8+ T cells [248]. In addition, during anti-TNF therapy, there is proliferation of the part of T regulatory cells [249,250], which are associated with greater sensitivity to *M. tuberculosis* infection [251]. The possible negative effects of anti-TNF therapy on antituberculous immunity are schematically summarized in Figure 4.

Currently, five TNF blockers are used in clinical practice: infliximab, adalimumab, certolizumab pegol, golimumab, and etanercept. Infliximab is a chimeric anti-TNF monoclonal antibody, consisting of a human Fc fragment of IgG1 and a mouse variable fragment. Adalimumab and golimumab are humanized monoclonal anti-TNF antibodies. Certolizumab pegol is a pegylated humanized anti-TNF Fab’ fragment. Etanercept consists of two human TNFR2 extracellular domains linked to the human Fc fragment of IgG1 [236,239]. Etanercept binds only the trimeric soluble form of TNF, while infliximab and adalimumab bind both the trimeric and monomeric soluble forms of TNF [236]. Infliximab complexes with soluble and membrane forms of TNF are more stable compared to etanercept and TNF complexes. Etanercept binds the soluble and membrane forms of TNF in a 1:1 ratio, whereas infliximab and adalimumab can bind both mediator forms in a 2:1 ratio [236,252]. Differences in the structure of TNF inhibitors and their biological activity lead to differences in their effectiveness, regimen, and the incidence of side effects of TNF blockade, including the development of TB infection [239,253].

When infliximab is used in the therapy of autoimmune diseases, TB infection develops in some patients after 11–12 weeks of treatment [237].

Infliximab, adalimumab, and etanercept inhibit maturation of mycobacterium-containing phagosomes in the cell line, but in the primary culture of monocyte-derived macrophages, only the use of anti-TNF antibodies leads to impaired phagosome maturation. Probably, differences in the inhibition of phagosome maturation by preparations based on the soluble TNF receptor and anti-TNF antibody are related to differences in the effects of these drugs on the membrane form of TNF [236].

TNF inhibitors induce apoptosis of immune cells, which are necessary for an immune response against *M. tuberculosis*; in addition, as a result of the death of CD8+ cells mediating the death of infected macrophages, inhibition of pathogenic bacteria elimination occurs. It has been established that during infliximab therapy, apoptosis of activated T cells occurs [254]. Infliximab induces the death of *M. tuberculosis*-specific CD8+ CCR7- CD45RA+ effector memory T cells via a surface-expressed membrane form of TNF by way of complement-dependent cytotoxicity, which leads to a decrease in the amount of perforin and granulisin, needed for the death of infected macrophages and pathogens, respectively [248]. In cell line studies, it has also been shown that infliximab causes cell apoptosis by reverse signal transduction via a membrane form of mediator, while etanercept has no such effect [255,256].

TNF blockers can inhibit T cell activation and secretion of IFN-γ, which are key for protection not only against *M. tuberculosis*, but also against other intracellular pathogens, in both in vitro and in vivo experiments [257,258,259,260]. Infliximab and adalimumab inhibit the proliferation and activation of CD4+ T cells and their production of IFN-γ mediated by *M. tuberculosis*, while etanercept has no such effect [258,259]. Infliximab reduces *M. tuberculosis*-stimulated proliferation of γδ T cells, which can lyse *M. tuberculosis*-containing macrophages and are an early source of IFN-γ production [261], and reduces expression of TNFR2 and IFN-γ by γδ T cells [262].

TNF blockers can also affect T regulatory cells, which have an important role in the immunopathogenesis of TB infection. On the one hand, they inhibit the immune response against *M. tuberculosis* [251]; on the other hand, they limit the excessive pro-inflammatory response, thereby reducing tissue damage [239]. It is likely that a balance between the pro-inflammatory response mediated by monocytes and macrophages and effector T cells and the anti-inflammatory effects of T regulatory cells is necessary for the formation and maintenance of the correct granuloma structure. The use of infliximab leads to the restoration of the ability of T regulatory cells in patients with autoimmune diseases to inhibit the production of pro-inflammatory mediators TNF and IFN-γ by activated T cells and monocytes, and to induce the formation of an immunosuppressive phenotype of CD4+ CD25+ T cells [250]. In addition, infliximab leads to the induction of CD4+ CD25high FoxP3+ CD62L- T regulatory cells, which have greater suppressive activity against T cell proliferation and pro-inflammatory cytokine secretion, unlike CD4+ CD25high FoxP3+ CD62L+ T regulatory cells [249]. It is likely that when administering TNF inhibitors, the formation of a T regulatory cell population with more pronounced immunosuppressive properties can lead to a weakening of the immune response in TB infection.

It is necessary to apply and develop approaches to anti-cytokine therapy using blockers of other mediators, the decrease in the biological activity of which is not at risk of TB progression [263]. In addition, an approach has been proposed to inhibit TNF that would not block the protective properties of TNF in intracellular infections. It involves the use of a TNF inhibitor, which is binding to a soluble but not membrane form of TNF [264]. At the same time, it has been shown that the membrane form of TNF is sufficient to control BCG infection and acute *M. tuberculosis* infection, but a soluble form of TNF is also necessary to control chronic *M. tuberculosis* infection [265].

It is important to note that the risk of reactivation of TB and other infectious granulomatous pathologies with the use of TNF inhibitors is not very high; in addition, in the case of TB development and during infectious pathology treatment, including preventive measures, treatment with TNF blockers can be continued [239,266].

### 6.2. Anti-TNF Therapy in Autoimmune Disease and Tuberculosis Reactivation

The use of anti-TNF agents has significantly improved clinical outcomes in autoimmune and inflammatory diseases. However, these therapies also impair host immunity against *M. tuberculosis*, leading to a higher risk of tuberculosis reactivation, particularly in patients with latent infection [267].

The likelihood of reactivation varies depending on the structure and mechanism of the TNF inhibitor. Monoclonal antibodies such as infliximab and adalimumab bind both soluble and membrane-bound TNF, resulting in profound immunosuppression. In contrast, receptor-based inhibitors like etanercept primarily bind soluble TNF and are associated with a lower risk [268]. Among currently approved TNF inhibitors, monoclonal antibodies such as infliximab and adalimumab are associated with a higher risk of tuberculosis reactivation due to their ability to block both soluble and membrane-bound TNF; whereas etanercept, a soluble receptor fusion protein, demonstrates a comparatively lower risk, likely owing to its more limited impact on membrane-bound TNF signaling [267,268,269].

Several key immunological mechanisms contribute to the increased susceptibility observed during anti-TNF therapy. First, TNF is essential for the formation and maintenance of granulomas, which serve as immunological barriers to contain latent bacilli. Disruption of granuloma architecture under TNF blockade facilitates pathogen escape and dissemination. Second, anti-TNF treatment reduces perforin and granulysin expression in cytotoxic T cells, weakening direct antimicrobial activity. Third, TNF is required for proper phagosome maturation in macrophages; its inhibition leads to impaired bacterial killing [269].

These observations underscore the importance of TB risk mitigation in patients undergoing TNF blockade. Standard practice includes screening for latent tuberculosis infection using interferon-gamma release assays (IGRAs) or tuberculin skin testing (TST) prior to therapy [267]. In patients with evidence of latent infection, preventive therapy substantially reduces the incidence of reactivation and should be initiated according to national and international guidelines. Thus, while TNF inhibitors remain indispensable in the treatment of autoimmune conditions, their use must be balanced against the risk of TB reactivation. The mechanisms involved–ranging from granuloma destabilization to T cell dysfunction–highlight the central role of TNF in antimycobacterial defense.

## 7. The Role of the Membrane Form of TNF in the Immune Response to Tuberculosis

The contributions of soluble and membrane forms of TNF to host defense against mycobacterial infections have been extensively studied [265,270,271,272]. Membrane TNF alone can control acute infections caused by non-virulent BCG but is insufficient for long-term protection against virulent *M. tuberculosis* [271]. In BCG infection, mice expressing only membrane TNF show partial protection: their pathogen burden is lower than in TNF-deficient mice, though higher than in wild-type mice [271]. Structured granuloma formation is preserved in these mice, albeit with increased inflammation compared to wild-type controls [271].

Upon infection with virulent *M. tuberculosis*, membrane TNF supports survival during acute phases, similar to wild-type mice, yet fails to prevent disease progression during chronic infection, leading to increased lung pathology and eventual mortality [270,271]. Despite comparable bacterial loads early in infection, granulomas in membrane TNF mice exhibit abnormal organization and reduced numbers during chronic stages [270,271].

At the immunometabolic level, macrophages from membrane TNF mice display enhanced activation markers (MHC II, CD80, CD86), but iNOS expression is dispersed, indicating granuloma disorganization [265]. Altered cytokine profiles, including elevated IFN-γ and IL-10 and decreased IL-12, mirror features seen in TNF-deficient mice [270,273,274,275,276]. Moreover, NOS2 activation, crucial for pathogen control, is delayed yet ultimately higher during chronic BCG infection in membrane TNF mice compared to wild types [270,277,278].

The membrane form of TNF influences granuloma architecture by supporting the formation of smaller but well-differentiated granulomas composed of activated macrophages [270]. During BCG infection, membrane TNF mice initially display lower IFN-γ but higher IL-12p40 serum levels, with normalization at later stages [270,279,280]. Antigen-specific IFN-γ and NO production by splenocytes are reduced in these mice compared to wild types, highlighting partial impairments in T cell responses [270].

In tuberculosis infection, CD14^+^CD16^+^ and CD14^+^CD3^+^ monocytes expand and produce high levels of TNF, IL-1β, and other inflammatory mediators [281,282,283,284]. Particularly, CD3^+^TCRαβ^+^ macrophages express CD1 molecules, membrane TNF, and pro-inflammatory cytokines, linking lipid antigen presentation with TNF-mediated responses [284,285,286,287]. The membrane form of TNF promotes the proliferation of both CD3^+^TCRαβ^+^ and CD3^+^TCRαβ⁻ macrophages at infection sites [284].

BCG infection induces a receptor switch in CD3^+^ macrophages from TNFR1 to TNFR2 dominance, suggesting a transition from migratory to activation functions [284,288,289]. In TNF-deficient mice, exaggerated IFN-γ responses by CD4^+^ T cells are observed, while membrane TNF moderates antigen-specific T cell activation, reflecting its regulatory role in inflammatory responses [288].

Thus, while membrane TNF is sufficient for initial control of infection and granuloma organization, the absence of soluble TNF compromises long-term immunity against virulent *M. tuberculosis*.

## 8. Role of TNF of Myeloid and T Cell Origin in the Immune Response in Tuberculosis

Studies using mice with cell-specific *TNF* gene inactivation demonstrated that TNF produced by myeloid cells (macrophages and neutrophils) is crucial for early control of *M. tuberculosis* infection, while T cell-derived TNF is essential for maintaining protective immunity during chronic infection [290,291]. Myeloid cell-derived TNF regulates bacterial growth, inflammation, and immune cell recruitment into the lungs during early infection but is not required for granuloma organization [290]. In contrast, T cell-derived TNF is critical for preserving granuloma structure, limiting necrosis, and maintaining alveolar integrity during chronic stages of TB [290]. Mice deficient in T cell TNF develop extensive necrosis without defined granulomas, leading to widespread lung damage. Deficiency of both myeloid and T cell TNF reproduces the phenotype observed in complete TNF knockout mice, underscoring the complementary roles of both sources of TNF in TB immunity [290,292].

## 9. Effect of Drug-Resistant M. tuberculosis Strains on TNF-Mediated Immunity

*M. tuberculosis* strains associated with multidrug-resistant (MDR) TB modulate the host immune response differently compared to drug-sensitive strains. MDR strains reduce TNF and IL-8 secretion by pulmonary epithelial cells, suppress macrophage apoptosis and cytotoxicity, decrease reactive oxygen species production, and inhibit chemokine secretion by CD8+ T cells, facilitating their survival within the host [124,293,294,295]. Although preincubation of macrophages with MDR strains decreases apoptosis, it does not significantly alter TNF production upon subsequent exposure to drug-sensitive strains [293]. Furthermore, MDR strains do not affect TNFR1 and TNFR2 expression, and the reduced macrophage apoptosis they induce is not linked to increased IL-10 production [293].

## 10. Dual Role of TNF in Protection and Pathogenesis

While TNF is vital for mounting an effective immune response against TB, its excessive activity can also contribute to disease pathology. It may promote tissue necrosis, facilitate *M. tuberculosis* replication in monocytes, and exacerbate inflammatory damage [128,137,296,297,298]. Notably, in monocytes incubated with TNF, an increase in *M. tuberculosis* proliferation has been observed, but this effect can be reversed by the addition of iron compounds, which suppress bacterial growth and concurrently reduce TNF production by monocytes [297]. A similar inhibitory effect is seen when TNF is removed from the culture after preincubation, suggesting that prolonged TNF signaling may favor bacterial survival [297]. Moreover, the ability of different mycobacterial strains to induce TNF production correlates with their virulence: virulent H37Rv induces high TNF levels and a greater proportion of infected monocytes, BCG induces moderate TNF and infection levels, while avirulent H37Ra elicits low TNF production and fewer infected cells [128]. Several characteristic symptoms of TB infection—such as fever, weight loss, anorexia, and tissue damage—resemble the systemic effects of elevated pro-inflammatory cytokines and may, in part, result from the pathological activity of TNF [6]. In mice, depletion of B cells during the chronic phase of TB infection (weeks 12–16) is associated with severe lung inflammation, cytokine overexpression (IL-1, IL-11, IL-17A, TNF, IL-6), and worsened clinical outcomes, including cachexia and reduced survival [299].

Excessive TNF production during chronic *M. tuberculosis* infection has been shown to disrupt granuloma structure, leading to necrosis and loss of containment function [300]. This disorganization facilitates the extracellular release and dissemination of *M. tuberculosis*, thereby contributing to increased disease severity and systemic inflammation [301]. In parallel, high TNF levels are associated with immune dysregulation, including depletion of protective T cell subsets and impaired immunological control of persistent infection [302].

Tuberculous granulomas limit *M. tuberculosis* dissemination; however, disrupted granuloma structure can lead to tissue destruction and cavity formation. In pulmonary TB patients with cavities, elevated TNF levels in bronchoalveolar fluid are associated with necrosis, enhanced collagenase activity, reduced collagen synthesis, and cytotoxic effects on epithelial cells [303], contributing to lung damage [304].

In granulomas in which necrosis takes place, TNF levels are higher compared to those in which necrosis does not take place [305]. An excessive TNF level due to the activation of RIP5 kinase and its substrate PGAM5 leads to increased levels of mitochondrial reactive oxygen species (mROS)—superoxide and hydrogen peroxide in macrophages infected by mycobacteria [306,307]. mROS activate signaling pathways in organelles, including lysosomes and endoplasmic reticulum, which causes increased levels of calcium in mitochondria, leading to the development of macrophage necrosis and the release of mycobacteria into the intercellular space favorable for their reproduction [306,307]. However, an elevated TNF level does not lead to necrosis development in uninfected macrophages [131]. TNF can contribute to the necrosis of infected macrophages by enhancing glutaminolysis and succinate accumulation, which, in turn, promotes mitochondrial ROS (mROS) formation via reverse electron transport (RET) [131,308]. Mycobacterial factors further amplify this pathway, linking metabolic reprogramming to TNF-induced necrosis. Some studies suggest that drugs such as metformin may limit mROS formation and enhance anti-TB therapy, although results remain inconsistent [309,310,311].

Shedding of TNF receptors contributes to the downregulation of TNF activity in TB by sequestering the cytokine in soluble receptor complexes, and this mechanism is exploited by virulent mycobacterial strains to evade host immunity [179,312]. Mycobacterium-induced shedding of TNF receptors depends on the degree of virulence: infection with the avirulent *M. tuberculosis* strain H37Ra and attenuated BCG is accompanied by significantly lower levels of soluble TNF receptors compared to the virulent *M. tuberculosis* strain H37Rv [179,241,313,314]. Signal transduction from TNF via TNFR1 has key significance for the protective response in TB; however, in experiments in mice with a mutant form of TNFR1, which does not undergo shedding, it was shown that such animals were able to control acute *M. tuberculosis* infection but were unable to control the chronic infectious process [315]. Soluble forms of TNFR1 and TNFR2 regulate the biological activity of cytokine and control the IL-12-dependent migration of dendritic cells into lymph nodes, as well as the subsequent activation of Mtb-specific T cells [130,314].

## 11. TNF Anti-Inflammatory Activity

Although tumor necrosis factor (TNF) is best known for its pro-inflammatory properties, it also demonstrates important anti-inflammatory effects in the context of mycobacterial infections. These effects contribute to the fine-tuning of immune responses, preventing excessive inflammation and limiting tissue damage during persistent infection. The dual role of TNF underscores its complex immunoregulatory nature and its essential role in balancing protective immunity and immunopathology.

Experimental studies have demonstrated that TNF deficiency or its neutralization leads to excessive granulomatous inflammation and disorganized granulomas in late-stage mycobacterial infection [6,288]. This is associated with heightened IL-12 and IFN-γ production, suggesting a regulatory role of TNF in limiting Th1-driven inflammation [288]. One mechanism of this anti-inflammatory effect involves membrane-bound TNF, which supports the function of myeloid-derived suppressor cells (MDSCs) in BCG infection. Through TNFR2 on T cells, membrane TNF enables MDSCs to suppress CD4^+^ T cell proliferation and reduce IL-2 and IFN-γ secretion. In the absence of TNF signaling, this control is lost, leading to uncontrolled T cell activation [288].

In addition to MDSC-mediated immunoregulation, membrane TNF contributes to the maintenance of granuloma integrity and promotes the structured recruitment of immune cells, thereby localizing and limiting the inflammatory response [300,316]. These effects are distinct from those mediated by the soluble TNF, which is more strongly associated with systemic inflammation. Selective inhibition of the soluble form of TNF, while preserving membrane-bound TNF, has been proposed as a therapeutic approach to reduce TNF-associated tissue damage without impairing host defense [317]. Notably, during anti-TB therapy, both drug-sensitive and drug-resistant TB patients exhibit a decrease in TNFR1 and TNFR2 expression on CD4^+^ T cells; however, only individuals with drug-resistant TB maintain elevated TNF and TNFR2 transcript levels, suggesting persistent immune activation despite treatment [318].

Furthermore, TNF can induce the expression of anti-inflammatory mediators such as A20 (TNFAIP3), a ubiquitin-editing enzyme that dampens NF-κB signaling and modulates the intensity and duration of cytokine responses [319]. Through such mechanisms, TNF helps to prevent immune overactivation and contributes to immune homeostasis in chronic infection.

Taken together, these findings indicate that TNF, despite its well-characterized pro-inflammatory effects, also plays an important role in suppressing excessive immune responses and preserving lung tissue during the prolonged course of mycobacterial infection. This duality emphasizes the need for nuanced therapeutic strategies that can selectively modulate TNF pathways without compromising immune protection.

## 12. Autoantibodies to TNF in Tuberculosis

Autoantibodies to cytokines play an important role in the regulation of the biological activity of cytokines in normal and pathological conditions [320]. In TB, the presence of autoantibodies to IFN-γ [321], TNF, IL-17A, IL-31, and other autoantigens was shown [322]. In patients with active pulmonary TB, there are increased levels of autoantibodies to TNF of the IgG class and IgG3 subclass compared to healthy donors. In patients with pulmonary TB with an advanced infectious process, the level of anti-TNF autoantibodies of the IgG class and subclasses of IgG1 and IgG3 is higher compared to patients with a limited infectious process [117]. An increase in the content of autoantibodies to TNF of the IgG1 and IgG3 subclasses in patients with active pulmonary TB compared to healthy donors, as well as in patients with an advanced infectious process compared to patients with a limited infectious process, may indicate the active participation of autoantibodies to TNF in the activation of the complement system. This is because immunoglobulins of the IgG1 and IgG3 subclasses are the foremost in terms of their ability to interact with the complement component C1q. In contrast, IgG2 is capable of weak binding, while IgG4 is not capable of binding with C1q [323]. It is known that the complement system takes part in the protective immune response in *M. tuberculosis* infection [324]. Taking into account that in patients with pulmonary TB, the level of autoantibodies to TNF of the IgG class and IgG3 subclass is higher compared to healthy individuals and in patients with an advanced infectious process compared to patients with a limited process, this may indicate a damaging role of these antibodies in pathology [325]. This effect may be due to an increase in the biological activity of TNF as a result of the formation of cytokine autoantibody complexes, which bind to Fc receptors and cause the production of pro-inflammatory mediators [325]. In addition, some antibodies promote an increase in the elimination half-life of cytokines, acting as a reservoir (carrier) [326,327], also playing a significant role in the immune response in active pulmonary TB.

## 13. Tuberculosis Cases in Patients with TNF Deficiency

Two related patients in Colombia with recurrent TB were found to carry a loss-of-function mutation in the TNF gene, resulting in a complete absence of TNF secretion, even upon stimulation with IFN-γ or BCG [328]. Despite preserved TNFR1 and TNFR2 expression, and normal levels of IFN-γ and other cytokines, the patients’ GM-CSF-stimulated macrophages exhibited a marked reduction in reactive oxygen species production in response to BCG and *M. tuberculosis*. Both patients displayed normal counts of lymphoid and myeloid cells, formed granulomas, and developed typical inflammatory symptoms, including fever, elevated markers, and septic shock in listeriosis [67,329]. These cases illustrate that although some inflammatory responses and granuloma formation may occur independently of TNF, the cytokine is essential for the proper activation and antimicrobial function of alveolar macrophages during mycobacterial infection [328].

## 14. Summary and Perspectives

TB remains one of the major global health challenges. Despite growing investments in the study of its pathogenesis, as well as efforts to improve existing treatment strategies and develop new ones, the incidence and mortality rates associated with TB remain high.

TNF and its receptors—particularly TNFR1—play a critical role in the host’s protective immune response to *M. tuberculosis*. They participate in several essential mechanisms of infection control and bacterial clearance. These include the induction of apoptosis in infected macrophages, the maturation of dendritic cells, the immunometabolic reprogramming of macrophages, the stimulation of chemokine and adhesion molecule production, and the formation and maintenance of granulomas. At the same time, TNF can also contribute to pathological processes. It has been shown to support the intracellular replication of *M. tuberculosis* in monocytes and to promote tissue damage, granuloma necrosis, and systemic symptoms such as fever, weight loss, and anorexia. Thus, the role of TNF and its receptors in TB protection is multifaceted and complex. It is necessary to understand where the balance lies between the protective and damaging effects of TNF in this pathology: the balance between the enhancement of the protective effects of TNF and inhibition of the mechanisms mediated by them, contributing to the progression of infection and the development of cachexia. In addition, it is necessary to continue the search for approaches to the selective inhibition of the soluble TNF form in autoimmune diseases in order to prevent the development of most of the side effects of anti-TNF therapy. It is important to investigate further the role of TNF, produced by different cell types during the immune response in TB. It is important to study the advantages and disadvantages of the existing animal models of TB infection and select the most adequate ones, as well as search for new animal models, which can help in the study of various aspects of human TB pathogenesis and the role of TNF in the immune response in this disease. A comparison of the immune response parameters during infection with different mycobacteria, as well as *M. tuberculosis* isolates with different pathogenicity, will also contribute to the investigation of TB pathogenesis and the TNF role in the immune response in TB infection. Thus, the study of the role of TNF in TB and its relationship with various aspects of immunopathogenesis is necessary for the development of new approaches to the therapy of this nosology.

Based on the accumulated evidence, several important directions for future research can be outlined. These include further investigation of the cell type-specific functions of TNF during the immune response to *M. tuberculosis*, with particular attention to the contributions of myeloid, lymphoid, and stromal cells. The role of soluble versus membrane-bound TNF forms, and their differential effects in infection control and immunopathology, remains an area of significant interest, particularly in the context of developing more selective immunomodulatory therapies.

In addition, it is relevant to continue the search for and comparative evaluation of animal models that accurately reproduce the human immune response to *M. tuberculosis* and TNF-related mechanisms, as this will improve the translational value of preclinical findings.

Another promising avenue is the study of strain-specific effects of *M. tuberculosis* on TNF production and signaling, which may provide insight into mechanisms of virulence and immune evasion. Expanding our understanding of these aspects may ultimately contribute to the identification of novel therapeutic targets aimed at preserving the protective functions of TNF while minimizing its pathological consequences.

## Figures and Tables

**Table 1:** Summary of TNF-mediated effects in tuberculosis.

Function	Cellular Targets	Mechanism	Biological Outcome
Initiation of Inflammatory Response	Endothelium, macrophages, dendritic cells	Induction of IL-1, IL-6, IL-8, GM-CSF, increased permeability and adhesion molecule expression	Recruitment of immune cells to the infection site
Activation of Innate Immunity	Macrophages, neutrophils, DCs	Enhances ROS production, phagocytosis, MHC-II, and co-stimulatory molecule expression	Efficient pathogen clearance
Maintenance of Granulomas	Macrophages, T cells	Sustains macrophage activation and cell recruitment within granulomas	Latent TB control, containment of *M. tuberculosis*
Adaptive Immunity Stimulation	T lymphocytes, APCs	Enhances migration, antigen presentation, chemokine secretion	Amplified Th1 response and bacterial killing
Apoptosis of Infected Cells	Macrophages, epithelial cells, infected APCs	TNFR1 signaling activates caspases via the death domain	Elimination of intracellular pathogen niches
Regulation of Systemic Inflammation	Systemic immune network	High TNF levels cause cytokine storms, and affect distant organs	Potential immunopathology: tissue necrosis, shock, multi-organ damage
Interaction with Other Cytokines	IFN-γ, IL-1β, IL-10, TGF-β pathways	Modulates expression and effects of synergistic (IFN-γ) and regulatory (IL-10, TGF-β) cytokines	Balanced immune response; dysregulation can lead to immunopathology or tolerance
Stimulation of MTB Growth (Pathological)	Monocytes	Inhibition of apoptosis, lysosome fusion; metabolic shift to glycolysis	Persistence and intracellular survival of *M. tuberculosis*
